# Determining the parameter space for effective oxygen depletion for FLASH radiation therapy

**DOI:** 10.1088/1361-6560/abe2ea

**Published:** 2021-02-25

**Authors:** B C Rothwell, N F Kirkby, M J Merchant, A L Chadwick, M Lowe, R I Mackay, J H Hendry, K J Kirkby

**Affiliations:** 1 Division of Cancer Sciences, Faculty of Biology, Medicine and Health, The University of Manchester, Manchester, United Kingdom; 2 The Christie NHS Foundation Trust, Manchester, United Kingdom; 3 Christie Medical Physics and Engineering, The Christie NHS Foundation Trust, Manchester, United Kingdom

**Keywords:** FLASH radiotherapy, dose rate, oxygen depletion

## Abstract

There has been a recent revival of interest in the FLASH effect, after experiments have shown normal tissue sparing capabilities of ultra-high-dose-rate radiation with no compromise on tumour growth restraint. A model has been developed to investigate the relative importance of a number of fundamental parameters considered to be involved in the oxygen depletion paradigm of induced radioresistance. An example eight-dimensional parameter space demonstrates the conditions under which radiation may induce sufficient depletion of oxygen for a diffusion-limited hypoxic cellular response. Initial results support experimental evidence that FLASH sparing is only achieved for dose rates on the order of tens of Gy s^−1^ or higher, for a sufficiently high dose, and only for tissue that is slightly hypoxic at the time of radiation. We show that the FLASH effect is the result of a number of biological, radiochemical and delivery parameters. Also, the threshold dose for a FLASH effect occurring would be more prominent when the parameterisation was optimised to produce the maximum effect. The model provides a framework for further FLASH-related investigation and experimental design. An understanding of the mechanistic interactions producing an optimised FLASH effect is essential for its translation into clinical practice.

## 1. Introduction

Radiotherapy is one of the most successful and cost-effective forms of cancer treatment (Cullen *et al* 2014), but is limited by the damage caused to healthy tissue surrounding the target tumour. Dose fractionation is routinely used to exploit the ability of healthy cells to recover better than cancerous cells. A typical treatment consists of several weeks of ∼1.8–3 Gy fractions, delivered 5 days a week in ∼10 min each. Recent interest in FLASH radiotherapy has led to the proposal of a new paradigm; the use of few or single fractions of ultra-high-dose-rate (>40–150+ Gy s^−1^) radiation (Favaudon *et al* 2014). It is suggested that FLASH may offer normal-tissue-sparing advantages which could reduce side effects for patients, shorten treatment times, eliminate need for motion control and facilitate dose-escalation strategies.

Ultra-high-dose-rate radiation was first explored in the 1960s–70s (Vozenin *et al* 2019, Hendry 2020), but was largely dismissed for radiotherapy because of uncertainty in knowing how best to deliver doses to tumours versus normal tissues (Berry 1973, Hendry 1979). Favaudon *et al* (2014) reawakened interest by demonstrating reduced pulmonary fibrosis in electron-FLASH irradiated mice compared to conventionally irradiated mice, while showing FLASH to be no less efficient in suppressing tumour growth. Follow-up studies demonstrated sparing of pig skin (Vozenin *et al* 2018), and of neurocognitive functions in mice after whole-brain irradiation (Montay-Gruel *et al* 2017, 2019). With growing interest in the clinical translation of FLASH, Bourhis *et al* (2019) recently treated a patient’s skin lesion using electron-FLASH radiation therapy.

Despite this, the complete mechanisms behind the FLASH effect are not yet fully understood (Durante *et al* 2018, Harrington 2018, Al-Hallaq *et al* 2019). The most prevalent explanation is that at ultra-high dose rates, oxygen is depleted by radiation-induced chemical interactions in cells already at low physiological oxygen tensions, at a rate that is too fast for re-oxygenation via diffusion from nearby blood vessels (Spitz *et al* 2019, Vozenin *et al* 2019). The induced state of transient severe hypoxia, and therefore radioresistance, thus becomes responsible for the observed sparing effect (Alper 1979). Early studies provided evidence for oxygen depletion in the form of characteristic ‘breakpoint’ survival curves which showed induced radioresistance at anoxic-like levels above a certain dose of ultra-high-dose-rate irradiation (Epp *et al* 1968, Nias *et al* 1969, Berry and Stedeford 1972, Weiss *et al* 1974). Similar decreases in radiosensitivity were observed for mouse intestine response, skin reactions in rat feet, and late lung injury in mice (Hendry 2020). It is also hypothesised that the normal tissue protection observed *in vivo* can be attributed to the sparing of naturally hypoxic stem cell niches (Pratx and Kapp 2019b). More recently, it was shown that an increase in local oxygen concentration in the brains of mice using carbogen breathing during irradiation reversed the neurocognitive sparing effects of FLASH, with further evidence demonstrating reduced levels of hydrogen peroxide (a reactive oxygen species) in FLASH irradiated aqueous solutions compared to conventionally irradiated solutions (Montay-Gruel *et al* 2019).

There is a lack of understanding of the potential sparing effects of oxygen depletion in tumour tissue (Hendry 2020). Hypoxia in tumours is induced as a diffusion-limited chronic effect caused by oxygen metabolism (consumption) producing oxygen gradients around isolated perfused vessels (Hall and Giaccia 2012). Hypoxia is also produced as a perfusion-limited acute effect, caused by changes in blood perfusion including complete vessel occlusion, resulting in changes in oxygenation in large numbers of cells (Hill and Bristow 2013). This present work uses a model based on the fundamental principles behind oxygen diffusion and depletion in tissue to explore how these processes in the diffusion-limited hypoxia scenario should be affected under FLASH irradiation.

Similar models have been used extensively for the investigation of oxygen kinetics within biological systems (Gerlee and Anderson 2010, Grimes *et al* 2014, Aleksandrova *et al* 2016). However, few have done so with the application of ultra-high-dose-rate irradiation. Work by Pratx and Kapp (2019a) has related simulated FLASH-induced oxygen depletion to oxygen enhancement of radiation damage. A key finding was that only cells somewhat hypoxic at the time of irradiation would exhibit the FLASH effect, which is consistent with previous *in vitro* and *in vivo* results (Vozenin *et al* 2019). This present work aims to expand upon some of the simplifications in their model, and explore additional variation of the parameters involved. Conversely, a study by Zhou *et al* (2020) used a dimensional analysis approach to determine that a clinically-observable change in radiosensitivity from FLASH-induced oxygen depletion would be possible for normal tissue given a minimum dose rate of ∼60 Gy s^−1^. It is clear then that, while oxygen depletion remains a widely-accepted explanation for FLASH, there is a lack of understanding of the parameters at play which influence the possibility of a sparing effect. The aim of this study is to provide a more comprehensive overview of these physical, chemical and biological parameters involved, and to demonstrate the model framework built to explore their impact on a potential FLASH effect.

## 2. Methods

### 2.1. The reaction-diffusion model

A typical reaction-diffusion model in a one-dimensional slab geometry is used. Fickian diffusion is combined with terms for metabolic and radiation-induced oxygen consumption to model the flux of oxygen through a system of cells normal to a source (or capillary). Use of a Krogh cylinder geometry is common in other models to approximate the shape of a capillary (Grimes *et al* 2014), however the radial-diffusion assumption adopted is equivalent to the approach used here. Assuming an isothermal system with a constant diffusivity (Crank 1975), the model is:

\begin{eqnarray*}\displaystyle \frac{\partial C}{\partial t}={D}_{{\mathrm{eff}}}\displaystyle \frac{{\partial }^{2}C}{\partial {x}^{2}}-r,\end{eqnarray*}

where *x* is position (in metres), *t* is time (in seconds), *C* is oxygen concentration (in mol m^−3^), *D*_eff_ is the effective diffusivity (in m^2^ s^−1^) and *r* is the overall rate of reaction (in mol m^−3^ s^−1^), defined as positive for destruction of oxygen (see table 1).

**Table 1.** Parameters used in the model with their definitions and symbols.

Symbol	Units	Definition
*D*_mol_	m^2^ s^−1^	Molecular diffusivity of oxygen

*τ*	None	Tortuosity (nonlinearity of the path traversed by diffusing molecules)

*ϵ*	None	Cellular voidage (fraction of volume not occupied by cells)

*D*_eff_	m^2^ s^−1^	Effective diffusivity of oxygen

*p*_0_	mmHg	Partial pressure of oxygen at *x* = 0 in model (at oxygen supply/capillary)

*C*_0_	mol m^−3^	Concentration of oxygen at *x* = 0 in model (at oxygen supply/capillary)

*C*(*x*, *t*)	mol m^−3^	Concentration of oxygen as a function of position and time

*L*	m	Length of the system (half the distance between adjacent capillaries)

*r*	mol m^−3^ s^−1^	Overall rate of oxygen consumption

*k*	mol m^−3^ s^−1^	Metabolic rate constant

*k*_*s*_	mol m^−3^	Metabolic saturation (Michaelis–Menten) constant

*k*_1_	mol m^−3^ Gy^−1^	Reaction rate constant for radiolytic species production

*k*_2_	mol m^−3^ s^−1^	Oxygen depletion rate constant for two-stage radiolytic model

${k}_{d}^{0}$	mol m^−3^ Gy^−1^	Oxygen depletion rate constant for zero-order radiolytic model

${k}_{d}^{1}$	Gy^−1^	Oxygen depletion rate constant for first-order radiolytic model

*D*	Gy	Dose

$\dot{D}$	Gy s^−1^	Dose rate

Δ*x*	m	Node width

Δ*t*	s	Time-step

For a system of length *L*, the boundary conditions are:

(i) At *t* = 0, for all *x* > 0, *C* = 0.
(ii) At *x* = 0, for all *t* ≥ 0, *C* = *C*_0_ (where *C*_0_ is the boundary concentration).
(iii) At *x* = *L*, $\tfrac{\partial C}{\partial x}=0$ (symmetry condition).

We assume a constant supply of oxygen from the capillary at *x* = 0, thereby ignoring the perfusion-limited hypoxia scenario. The symmetry condition accounts for the existence of an adjacent capillary, where the length of the system, *L*, is half the capillary separation. In contrast to the model by Pratx and Kapp (2019a), the impact of varying this separation can be investigated using this model.

Numerical methods must be employed to solve equation (1). This present work has used a cellular automaton—a solution technique used to reduce complex processes within continuous systems into a sequence of iterative time-steps applied to a discrete number of nodes (representations of small units of space) (Alarcón *et al* 2003, Richard *et al* 2009). Nodes are arranged in a grid and exist within a neighbourhood of surrounding nodes. For each time-step, the state of each node changes according to given rules or functions which depend on the current state of the node and its neighbours. A number of studies have used this technique to model oxygen diffusion in cells (Alarcón *et al* 2003, Gerlee and Anderson 2010, Paul-Gilloteaux *et al* 2017).

**Figure 1.**
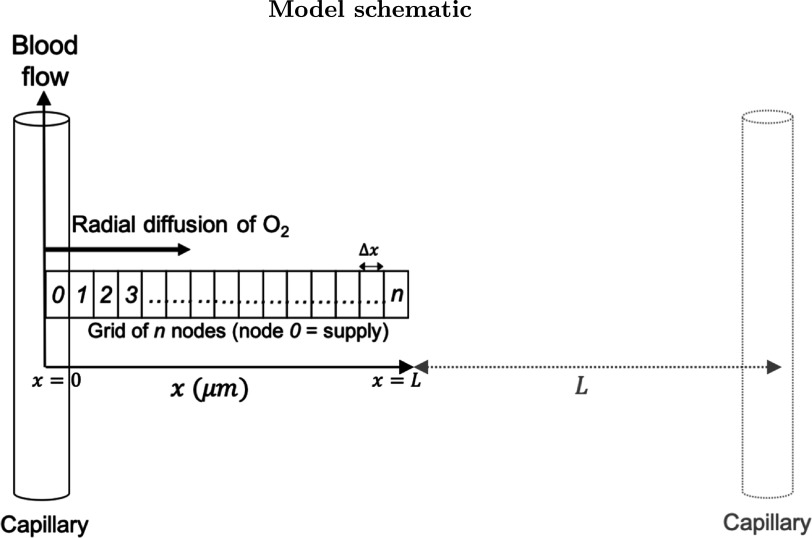
Schematic diagram of the model showing radial diffusion from a capillary along an orthogonal grid of nodes. An adjacent capillary is shown, where the distance between two capillaries is 2*L*.

Here a one-dimensional grid of nodes was used, each with a neighbourhood of two nodes and in a state characterised by its oxygen concentration. A schematic diagram is shown in figure 1. Alternating calculation steps of diffusion and reaction were applied at each time-step, as detailed below. Capillaries are modelled as ‘supply nodes’ which are reset after each set of time-steps to a specified concentration to simulate the continuous receiving of oxygen from the blood. In this present work, a single supply node at *x* = 0 was used to demonstrate the simplest case of diffusion from a single capillary. Checks within the model ensure the oxygen in the nodes has first built up to a steady state, where concentration remains approximately constant, before dose is applied. Nodes can then be irradiated for a duration specified by the dose and dose rate, and allowed to recover.

#### 2.1.1. Modelling diffusion

Contrary to previous oxygen depletion models (Pratx and Kapp 2019a), the effective diffusivity, *D*_eff_, is used in this present work to account for the porous nature of space between cells filled with an extracellular matrix. It is related to the molecular diffusivity, *D*_mol_, via the voidage, *ϵ*, which gives the fraction of volume not occupied by cells (i.e. accessible to diffusing oxygen) over the total volume, and the tortuosity, *τ*, which describes the non-linearity of the path traversed by diffusing molecules relative to an obstacle-free medium (Hrabe *et al* 2004):

\begin{eqnarray*}{D}_{{\mathrm{eff}}}=\displaystyle \frac{\epsilon {D}_{{\mathrm{mol}}}}{\tau }.\end{eqnarray*}

Generally as voidage reduces, tortuosity increases and so *D*_eff_ can easily be an order of magnitude smaller than *D*_mol_. Models which do not account for this may therefore overestimate oxygen tensions in their cellular systems.

Diffusion is modelled by the transfer of oxygen along the line of nodes. The relationship between the effective diffusivity and the fractional amount of oxygen, *f*, exchanged between adjacent nodes during each time-step was arrived at through equating the oxygen flux with Fick’s first law of diffusion to give:

\begin{eqnarray*}{D}_{{\mathrm{eff}}}=\displaystyle \frac{f{\left({\mathrm{\Delta }}x\right)}^{2}}{2{\mathrm{\Delta }}t},\end{eqnarray*}

where Δ*t* is the time-step (s) and Δ*x* is the node size (m). These must be chosen such that 0 < *f* ≤ 1, to prevent the solution becoming unstable. Checks in the model ensure this is the case before simulations are run.

#### 2.1.2. Modelling metabolic consumption

The model given in equation (1) becomes more complex with the incorporation of specific reaction processes. The metabolic reaction accounts for the continual oxygen consumption by the cells. Many similar diffusion and reaction models assume a constant metabolic reaction rate (Gerlee and Anderson 2010, Pratx and Kapp 2019a). However, there is a wide range of metabolic rates reported in the literature, with dependencies on cell type, function and biological status (Wagner *et al* 2011). Furthermore, additional checks must be in place to ensure the oxygen concentration does not become negative. This present work adopts a more complex approach using Michaelis–Menten-style kinetics to approximate the metabolic dependency on the oxygen concentration available:

\begin{eqnarray*}{r}_{{\mathrm{metabolic}}}=\displaystyle \frac{{kC}}{{k}_{s}+C},\end{eqnarray*}

where *k* is the maximum reaction rate (metabolic rate constant) and *k*_*s*_ is the concentration at which the reaction rate saturates (saturation constant). It is assumed here that when oxygen is abundant, cells consume it at a fixed rate, and when oxygen is deficient, the consumption rate is proportional to the concentration available, as observed in various tissue oxygen consumption studies (Tang 1933, Secomb *et al* 1993). This means the concentration will never fall below zero for an exact solution. If *k*_*s*_ ≪ *C*, the rate tends towards the maximum rate of consumption (*r* → *k*, zero-order kinetics), and when *k*_*s*_ ≫ *C*, the rate becomes linear with concentration ($r\to \tfrac{k}{{k}_{s}}C$, first-order kinetics). These assumptions are often used as justification for an analytically solvable metabolic consumption model (Grimes *et al* 2014, Aleksandrova *et al* 2016). Some studies suggest that a zero-order approach is in fact suitable down to very low oxygen tensions (Place *et al* 2017), however it is these low oxygen regions which we are interested in for hypoxia-related studies. This present work aims to provide a more general model, but at the expense of analytical tractability.

#### 2.1.3. Modelling radiolytic depletion

The other contribution to the overall reaction rate is the radiation-induced consumption of oxygen. Similar to the approach taken by Zhou *et al* (2020), this model uses a two-stage lumped reaction:

(i) During the first stage, radiation interacts with the cells to produce radiolytic species *A*:
  \begin{eqnarray*}{{\mathrm{H}}}_{2}{\mathrm{O}}+\mathrm{radiation}\longrightarrow \ {A}.\end{eqnarray*}
  The cell here is modelled as a water-like system, where species *A* accounts for the lumped products of the physico-chemical stage of the radiolysis of water (Draganic and Draganic 1971, Ling 1975, Zhou *et al* 2020). It is assumed that the concentration of *A* produced is proportional to the radiation dose (in Gy), such that
  \begin{eqnarray*}{r}_{A}\propto \dot{D},\end{eqnarray*}
  where *r*_*A*_ is the rate of production of *A* (in mol m^−3^ s^−1^) and $\dot{D}$ is the dose rate (in Gy s^−1^).
(ii) During the second stage, species *A* reacts with intracellular oxygen to produce species *B* via the 2nd-order chemical reaction (Ling 1975, Zhou *et al* 2020):
  \begin{eqnarray*}{A}+{{\mathrm{O}}}_{2}\longrightarrow \ {B},\end{eqnarray*}
  where species *B* is likely a very reactive peroxide-type compound (Draganic and Draganic 1971).

The overall rate of production of *A* and rate of consumption of oxygen are given by:

\begin{eqnarray*}{r}_{A}={k}_{1}\dot{D}-{k}_{2}[A]C\end{eqnarray*}

\begin{eqnarray*}{r}_{{{\mathrm{O}}}_{2}}=-{k}_{2}[A]C,\end{eqnarray*}

where *k*_1_ and *k*_2_ are the reaction rate constants for each stage in mol m^−3^ Gy^−1^ and m^3^ mol^−1^ s^−1^ respectively.

This two-stage approach reflects results from literature which show that the amount of oxygen consumed during a single irradiation increases with dose (Weiss *et al* 1974, Whillans and Rauth 1980, Michaels 1986). A radiolytic depletion rate that is zero (Pratx and Kapp 2019a) or first (Petersson *et al* 2020) order with oxygen concentration could also reflect these findings. Some studies have shown that the slope of oxygen depletion yield is independent of initial oxygen concentration (Weiss *et al* 1974). However, these experiments are limited and may not hold at low oxygen tensions (Weiss *et al* 1975). The zero- and first-order models are respectively given as:

\begin{eqnarray*}{r}_{{radiolytic}}^{0}={k}_{d}^{0}\dot{D}\end{eqnarray*}

\begin{eqnarray*}{r}_{{radiolytic}}^{1}={k}_{d}^{1}\dot{D}C,\end{eqnarray*}

where the respective radiolytic depletion constants, ${k}_{d}^{0}$ and *k_d_* ^1^, provide the constants of proportionality in each case (in units of mol m^−3^ Gy^−1^ and Gy^−1^ respectively). In the zero-order case, checks in the model were used to fix the concentration at zero if it became negative. All three of these approaches were investigated using this model.

#### 2.1.4. Modelling change in radiosensitivity

The model solution can determine key parameters for radiation-induced oxygen depletion. The impact of this on cellular radiosensitivity can also be investigated. Like other models (Pratx and Kapp 2019a, Petersson *et al* 2020), this present work has used the relationship between oxygen level and subsequent radiosensitivity from oxygen enhancement. The oxygen enhancement ratio (OER) is defined as the ratio of doses under hypoxic and aerated conditions required to achieve the same biological effect—its value typically varies between 2.5 and 3.5 for various biological endpoints (Hall and Giaccia 2012). To characterise the oxygen concentration dependence on the basis of the oxygen fixation hypothesis, Grimes and Partridge (2015) used the ratio of cell kill in oxic conditions to that under anoxia at a given dose. This ratio is called here the oxygen kill ratio (OKR). They showed that this algorithm could be expressed in terms of OER, as confirmed by others using relative radiosensitivity, with radiosensitivity defined as the log-fraction of cells killed per Gy of radiation (Pratx and Kapp 2019a) (see the supplementary material (available online at stacks.iop.org/PMB/66/055020/mmedia)). The relationship between OER and oxygen tension which fits well with experimental data (at 10 Gy) is given by:

\begin{eqnarray*}{\mathrm{OER}}(p)=1+\displaystyle \frac{{\phi }_{O}}{{\phi }_{D}}(1-{e}^{-\psi p}),\end{eqnarray*}

where *p* is the oxygen partial pressure (in mmHg), $\tfrac{{\phi }_{O}}{{\phi }_{D}}$ is the ratio of cells killed under by oxygen fixation to those killed directly, and *ψ* is a rate constant derived from first principles. Values of 1.6 and 0.26 mmHg^−1^ have been established for $\tfrac{{\phi }_{O}}{{\phi }_{D}}$ and *ψ* respectively (Grimes and Partridge 2015). This produces a typical radiosensitivity curve which increases with oxygen partial pressure up to ∼20 mmHg, before saturating at a maximum OER of ∼2.6 (Hall and Giaccia 2012). Other FLASH models have used the similar parameterisation by Alper and Howard-Flanders (1956) (e.g. Zhou *et al* 2020). The Grimes and Partridge model and its parameters are well-validated by more recent experimental data. While the traditional definition of OER as a ratio of doses is a function of cell survival endpoint, the model derived by Grimes is based on a ratio of cell kill for a given dose, verified by OER data calculated from slope ratios from the exponential regions of cell survival curves (Grimes and Partridge 2015). Here we assume the relationship between OER and oxygen partial pressure holds for the tested range of doses. Although the OKR version is unsuitable for use at high doses where it has a limiting value of unity (see supplementary details), in its OER form the model is applicable here and can hence be used to evaluate the decrease in radiosensitivity as a result of irradiation at varying dose rates.

The shift in OER (ΔOER) from steady-state during irradiation could be calculated for each node in the simulation. Various quantifiers, such as the average or maximum ΔOER across the nodes, can be specified and calculated by the model to indicate changes in radiosensitivity. The effect of a fixed amount of oxygen depletion on OER is considerably greater at partial pressures of ∼20 mmHg than at higher pressures, so the steady-state oxygen concentrations are important to consider. Using the maximum OER (under aerated conditions) of 2.6 and minimum OER (under anoxic conditions) of 1, the maximum ΔOER using this model is 1.6 (Grimes and Partridge 2015).

The differential effect observed experimentally between tumour and normal tissue irradiated at ultra-high dose rates is suggested to be attributed to initial tensions in severely hypoxic tumour tissue residing in the region where little or no further change in radiosensitivity is achievable, whereas mildly hypoxic normal tissue tensions have the potential to undergo a relatively large shift in radiosensitivity (Wilson *et al* 2020). The model could be used to investigate this hypothesis by simulating radiolytic-depletion for both tumour- and normal-tissue-specific input parameters.

### 2.2. Model parameters

A literature search was performed to determine biologically-relevant ranges to test for each parameter. Values of each parameter tested with references to their respective literature sources are summarised in table 2. Both dose and dose rate were varied, with values based on findings from recent *in vivo* FLASH studies (Favaudon *et al* 2014, Montay-Gruel *et al* 2017, Vozenin *et al* 2018) and comparable FLASH models (Pratx and Kapp 2019a). Doses between 2 and 50 Gy, and dose rates between 0.1 and 150 Gy s^−1^, were used to both capture conventional treatments and test up to and beyond possible FLASH dose and dose rate thresholds (Montay-Gruel *et al* 2017, Vozenin *et al* 2018, Bourhis *et al* 2019).

**Table 2.** Parameter values used in simulations.

Parameter	Values tested	References
Dose rate	0.1, 50, 150 Gy s^−1^	Favaudon *et al* (2014)
		Montay-Gruel *et al* (2017)
		Montay-Gruel *et al* (2019)

Dose	2, 15, 50 Gy	Vozenin *et al* (2018)
		Montay-Gruel *et al* (2017)
		Montay-Gruel *et al* (2019)

*D*_mol_	1, 2, 16 × 10^−9^ m^2^ s^−1^	Gerlee and Anderson (2010)
		Pratx and Kapp (2019a)
		Curcio *et al* (2007)
		Kirkpatrick *et al* (2003)

*ϵ*	0.2	Hrabe *et al* (2004)
		Curcio *et al* (2007)

*τ*	1.4	Hrabe *et al* (2004)
		Curcio *et al* (2007)

*p*_0_	20, 40, 80 mmHg	Pratx and Kapp (2019a)
		McKeown (2014)
		Skeldon *et al* (2012)
		Curcio *et al* (2007)
		Goldman and Popel (2000)
		Kirkpatrick *et al* (2003)

*k*	0.001, 0.01, 0.1 mol m^−3^ s^−1^	Aleksandrova *et al* (2016)
		Skeldon *et al* (2012)
		Curcio *et al* (2007)
		Goldman and Popel (2000)

*k*_*s*_	0.0001, 0.001, 0.01 mol m^−3^	Aleksandrova *et al* (2016)
		Skeldon *et al* (2012)
		Curcio *et al* (2007)
		Goldman and Popel (2000)

*k*_1_ (and ${k}_{d}^{0}$)	0.0001, 0.0005, 0.001 mol m^−3^ Gy^−1^	Weiss *et al* (1974)
		Pratx and Kapp (2019a)
		Michaels (1986)
		Whillans and Rauth (1980)

*k*_2_	10^6^, 10^7^, 10^8^ mol m^−3^ s^−1^	Draganic and Draganic (1971)
		Ling (1975)

${k}_{d}^{1}$	0.001, 0.01, 0.1 Gy^−1^	Weiss *et al* (1974)
		Pratx and Kapp (2019a)
		Michaels (1986)
		Whillans and Rauth (1980)

*L*	20, 30, 50, 100 *μ*m	Freitas (1999)
		Hall and Giaccia (2012)

The parameters which determine the steady-state oxygen concentration (and impact the recovery of oxygen after depletion) are the diffusivity, capillary oxygen tension and the metabolic parameters. Literature oxygen diffusivity values are generally molecular diffusion coefficients of ∼1–2 × 10^−9^ m^2^ s^−1^ (Kirkpatrick *et al* 2003, Curcio *et al* 2007) which neglect the voidage and tortuosity of their systems. Hrabe *et al* (2004) determined values of 1.4 and 0.2 for tortuosity and voidage respectively in the brain. Although these values may vary for different tissues, they were used as an initial estimate to convert molecular diffusion coefficients into effective diffusivity values. The impact of overestimating the extent of oxygen diffusion from using the molecular diffusion coefficient instead of the effective diffusivity was also investigated by omitting these conversion terms The capillary oxygen tension, *p*_0_, was approximated from venous (∼40 mmHg, McKeown 2014) and arterial (∼80 mmHg, Shapiro 1995) oxygen tensions, with lower values tested to approximate more hypoxic conditions. Values of oxygen partial pressure (in units of mmHg or %O_2_) were converted into concentrations of dissolved oxygen (in mol m^−3^) using Henry’s Law, with a constant of 0.0299 atm litre per mg (730 mmHg m^3^ mol^−1^) for oxygen dissolved in water at 35 °C (Blanch and Clark 1997). Although the solubility of oxygen can be significantly lower than this in aqueous solutions, this represents a safe worst-case scenario for a FLASH effect. Reported metabolic rates vary significantly (Wagner *et al* 2011), particularly given additional variation in use of zero-order (Alarcón *et al* 2003, Gerlee and Anderson 2010, Pratx and Kapp 2019a) or Michaelis–Menten (Goldman and Popel 2000, Curcio *et al* 2007, Aleksandrova *et al* 2016) forms Maximum metabolic rate constants are generally of the order ∼10^−2^ mol m^−3^ s^−1^ while saturation constants are ∼10^3^ mol m^−3^. Orders of magnitude above and below each case were explored.

The rate constant for the production of radiolytic species *A* has been measured as 0.0001–0.001 mol m^−3^ Gy^−1^ (depending on the medium or bacteria system employed) (Weiss *et al* 1974, 1975, Michaels 1986). This range was used to approximate values for *k*_1_ and, in the zero-order model case, ${k}_{d}^{0}$. For the first-order radiolytic model, a range of 0.001–0.1 Gy^−1^ was used to account for the dependency on oxygen concentration (of the order 0.01 mol m^−3^) and the additional uncertainty this contributes.

Literature values for the rate of the reaction between molecular oxygen and *A*, *k*_2_, also show a large degree of uncertainty. The very short timescale over which the depletion of oxygen occurs (≲10^6^ s, Zhou *et al* 2020) suggests very large rates, however the order can vary between 10^6^ and 10^9^ mol m^−3^ s^−1^ (Draganic and Draganic 1971, Ling 1975). The short timescale of this reaction also determines the maximum time-step size, Δ*t*, for numerical stability. The tenfold increase in simulation time as Δ*t* is reduced by an order of magnitude means there is a trade-off between accuracy and computational expense. Tests were performed to ensure that for each value of *k*_2_, the largest value of Δ*t* which gave numerical stability and produced results which matched those produced by the next smallest order (to a significant degree) was used. For *k*_2_ = 10^9^ mol m^−3^ s^−1^, the resulting run time became unfeasibly long to perform multiple simulations; the initial parameter search was hence only performed for *k*_2_ up to 10^8^ mol m^−3^ s^−1^. The node width remained fixed for all simulations at 1 *μ*m.

The number of nodes is used to approximate half the distance between adjacent capillaries. Although this can vary significantly in tissue, the mean separation of 40 *μ*m (20 nodes) was used for the simulations in the example parameter space (Freitas 1999). Tests were also performed using 50 and 100 nodes to compare with other models (Pratx and Kapp 2019a), and to model the approximation that oxygen concentration becomes effectively zero in a space of ∼100 *μ*m from a source (Hall and Giaccia 2012).

To demonstrate the impact of some of the model parameters, an example parameter space was produced using three values each of the diffusivity, capillary tension, metabolic constants, radiolytic constants, dose and dose rate (6561 simulations in total). This is shown using a parallel coordinates plot, which represents relationships between highly multi-variate data (Watts and Crow 2019). The average shift in OER across the nodes for each parameter combination was calculated. Interactive plots allowed variable axes to be rearranged (to more easily derive patterns between different variables) and constraint ranges to be drawn (to isolate sections of data) to aid in analysis.

## 3. Results

### 3.1. Steady states

**Figure 2.**
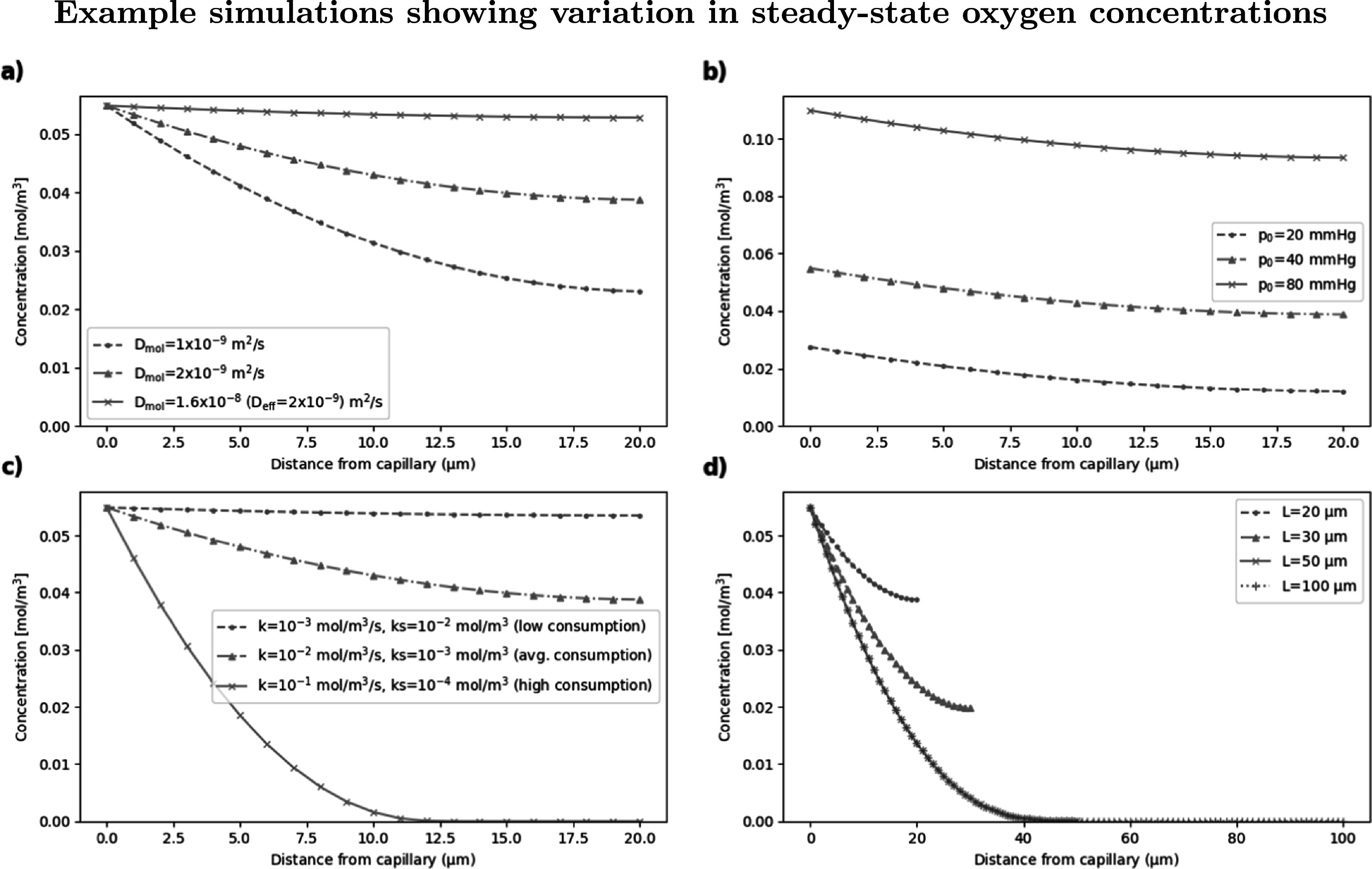
Example simulations showing steady-state oxygen concentration as a function of position for (a) different values of diffusivity; (b) different capillary tensions; (c) different values of metabolic parameters; and (d) different system lengths. Non-variable parameters in each case remained fixed at *D*_mol_ = 2 × 10^−9^ m^2^ s^−1^, *p*_0_ = 40 mmHg, *k* = 0.01 mol m^−3^ s^−1^, *k*_*s*_ = 0.001 mol m^−3^ and *L* = 20 *μ*m where necessary.

The variation in steady-state oxygen concentrations calculated from parameter values from different literature sources (see table 2) is demonstrated in figure 2. Since the steady-state parameters determine the initial oxygen levels before dose is applied (as well as the speed of recovery during and after dose), they have a strong impact on the change in radiosensitivity available for a given level depletion.

Figure 2(a) shows the impact of changing the diffusivity parameter (while keeping *L*, *p*_0_, *k* and *k*_*s*_ constant at 20 *μ*m, 40 mmHg, 0.01 mol m^−3^ s^−1^ and 0.001 mol m^−3^ respectively), where values of *D*_mol_ were converted to effective diffusivities as detailed above. Literature values of molecular diffusivity range between 1–2 × 10^−9^ m^2^ s^−1^ (Kirkpatrick *et al* 2003, Curcio *et al* 2007); although this variation is relatively small, the impact of the difference on the oxygen concentration is apparent. A molecular diffusivity of 1.6 × 10^−8^ m^2^ s^−1^ corresponds to an effective diffusivity of 2 × 10^−9^ m^2^ s^−1^, so reflects the case when the molecular diffusivity is treated as the effective diffusivity in tissue (Pratx and Kapp 2019a). As shown, this can result in a significant overestimation of the amount of oxygen available.

Figures 2(b) and (c) show the impact of varying the capillary tension and the metabolic parameters respectively (while keeping *D*_mol_ fixed at 2 × 10^−9^ m^2^ s^−1^). The combination of metabolic parameters, *k* and *k*_*s*_, were varied within the literature ranges to provide extreme and average cases of oxygen consumption. These parameters can have significant variation for different cells in the body (Wagner *et al* 2011), so it is important to consider this impact when attempting to model where and under what circumstances a FLASH sparing effect may occur. The system length is varied in figure 2(d), demonstrating the significance of the boundary condition at *x* = *L* outlined above.

### 3.2. Comparing radiolytic models

**Figure 3.**
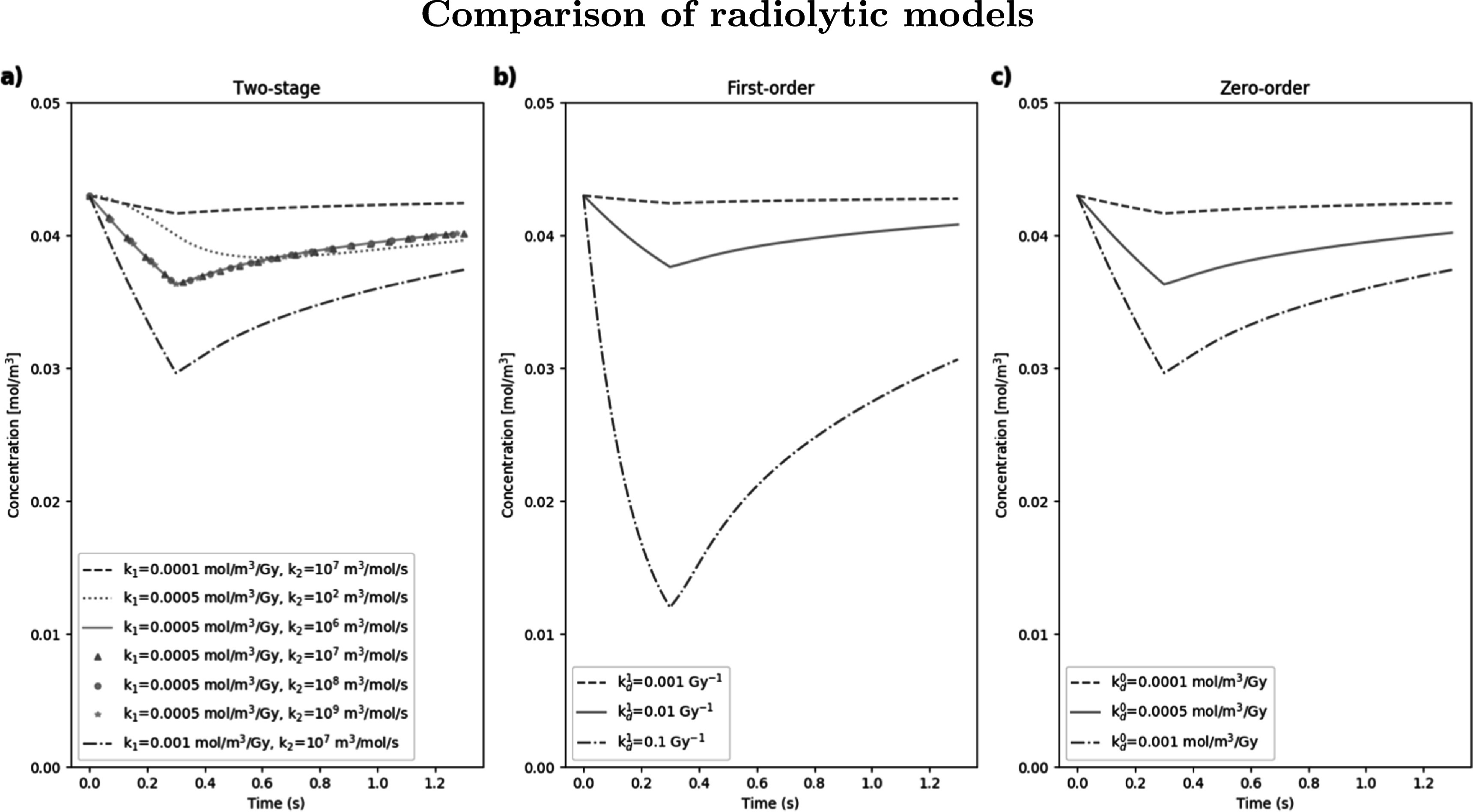
Example simulations showing oxygen concentration as a function of time for node 10, using (a) the two-stage radiolytic depletion model, with different values of *k*_1_ and *k*_2_; (b) the first-order model with different values of ${k}_{d}^{1}$; (c) the zero-order model with different values of ${k}_{d}^{0}$. Dose and dose rate remained fixed at 15 Gy and 50 Gy s^−1^ respectively for each simulation. Steady-state parameters remained at fixed values specified previously.

Simulations were run using each radiolytic model discussed, using various values of the depletion constants for each case. The dose and dose rate were kept constant at 15 Gy and 50 Gy s^−1^ respectively (demonstrating a ‘FLASH’ case), and the same steady-state parameters (*D*_mol_ = 2 × 10^−9^ m^2^ s^−1^, *p*_0_ = 40 mmHg, *k* = 0.01 mol m^−3^ s^−1^ and *k*_*s*_ = 0.001 mol m^−3^) were used for each run. Plots showing oxygen concentration as a function of time for the two-stage, zero-order, and first-order models are given in figure 3. An additional simulation using a very low value for *k*_2_ (10^−2^ m^3^ mol^−1^ s^−1^) was added to figure 3(a) to show how results vary as the order of magnitude of *k*_2_ approaches that of *k*_1_ (in their respective units).

From figures 3(a) and (c), it is clear that the two-stage and zero-order models are comparable for equal values of *k*_1_ and ${k}_{d}^{0}$ respectively (for *k*_2_ ≥ 10^6^ m^3^ mol^−1^ s^−1^). Although a zero-order approach may not be appropriate at very low oxygen concentrations, the results for this example generally agree with literature claims that rate of depletion appears independent of the initial oxygen concentration (Weiss *et al* 1974). The first-order approach in 3(b) may significantly overestimate the level of oxygen depletion for comparable values of the depletion parameter (Petersson *et al* 2020). The overlap of lines in figure 3(a) for equal values of *k*_1_ shows that, for the feasible range of *k*_2_ values tested (based on the variation in literature), there is almost no difference, as *k*_1_ is the driving parameter. When *k*_2_ ≫ *k*_1_, the second stage of the radiolytic depletion effectively becomes instantaneous, causing the two-stage model to be reduced to the zero-order case where oxygen depletes proportionally to the dose rate. This was tested for smaller *k*_2_ values and a divergence was only apparent at *k*_2_ ≲ 10^2^ m^3^ mol^−1^ s^−1^, significantly below the literature range (table 2). For this value, the second stage of the radiolytic depletion can no longer be approximated as instantaneous compared to the first, and the zero-order model is no longer a reasonable assumption.

### 3.3. Comparing dose rates

**Figure 4.**
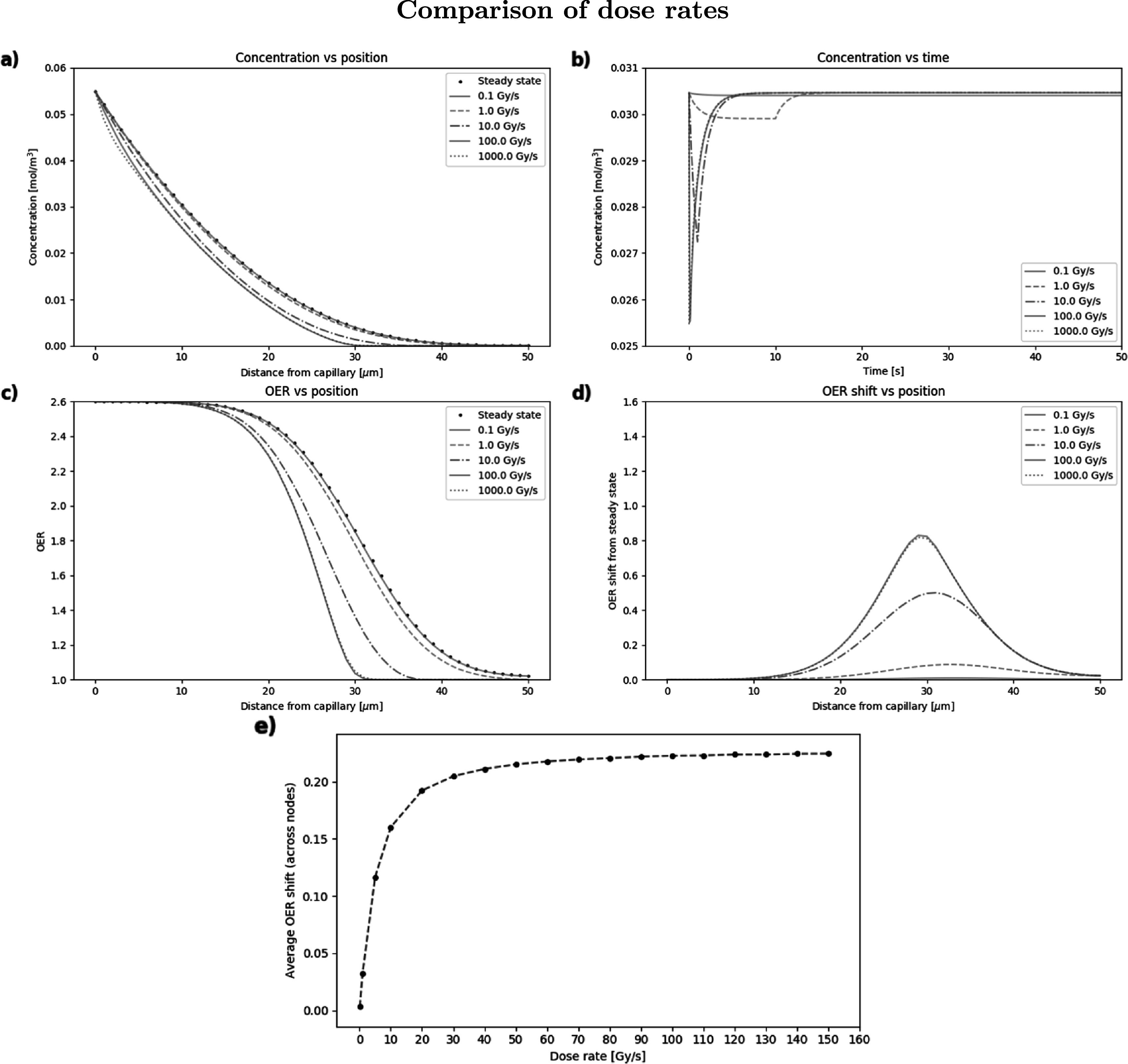
Example simulation showing for, different dose rates, (a) oxygen concentration as a function of position at steady-state and when the full dose has been applied; (b) concentration as a function of time for node 10; (c) OER at steady-state and dose-point as a function of position; (d) the difference between OER at steady-state and dose-point at each position, and (e) the OER shift from steady state averaged across the nodes as a function of dose rate. A dose of 10 Gy and depletion constants of *k*_1_ = 0.0005 mol m^−3^ Gy and *k*_2_ = 1 × 10^7^ m^3^ mol^−1^ s^−1^ were used for each simulation.

Figure 4 shows example simulations for dose rates from conventional up to and beyond FLASH values (0.1–1000 Gy s^−1^), to demonstrate some of the measurements that can be made with the model and how these change with dose rate. Oxygen concentration is shown as a function of position (at both steady-state and the point at which the total dose has been applied) and time (for node 10) in figures 4(a) and (b) respectively. Values of OER calculated at steady-state and dose-point (when the full dose was applied) are shown as a function of node position in figure 4(c), with the shift between these two timepoints given in figure 4(d) for each dose rate. The OER shift from steady state was averaged across the nodes and plotted in figure 4(e) as a function of dose rate. Further dose rates were measured here to investigate the smaller interval around the supposed FLASH threshold. These examples were run using *D* = 10 Gy, *D*_mol_ = 2 × 10^−9^ m^2^ s^−1^, *p*_0_ = 40 mmHg, *k* = 0.01 mol m^−3^ s^−1^, *k*_*s*_ = 0.001 mol m^−3^, *k*_1_ = 0.0005 mol m^−3^ Gy^−1^, *k*_2_ = 1 × 10^7^ m^3^ mol^−1^ s^−1^ and 50 nodes for each dose rate.

From this combination of parameters, there is a significant increase in ΔOER as the dose rate increases up to 100 Gy s^−1^, while almost no gain in ΔOER is achieved from increasing dose rate up to 1000 Gy s^−1^, as shown in figure 4(d). This is corroborated by figure 4(e), where the average ΔOER across the nodes begins to saturate at a dose rate of ∼50 Gy s^−1^, with only small further increases up to 150 Gy s^−1^. This may support claims from recent FLASH studies that a supposed dose rate threshold lies within this range (Vozenin *et al* 2018, Zhou *et al* 2020). At a dose rate of 0.1 Gy s^−1^, there is almost no change in oxygen concentration or OER from steady state, supporting the lack of sparing effect observed at conventional dose rates. Example calculation results for the average and maximum OER shifts across the nodes for each dose rate are given in table 3. Zhou *et al* (2020) predicted a minimum relative change in radiosensitivity of 3% for a clinically observable effect; from this example, this is obtained at dose rates ≳10 Gy s^−1^.

**Table 3.** Example OER shift calculations for different dose rates.

Dose rate (Gy s^−1^)	Avg ΔOER	Max ΔOER	Max ΔOER position (*μ*m)
0.1	0.0033 (0.2%)	0.0087 (0.6%)	33

1	0.033 (2.2%)	0.086 (5.7%)	33

10	0.16 (9.5%)	0.50 (30%)	31

50	0.21 (12.2%)	0.77 (42%)	30

100	0.22 (13%)	0.83 (44%)	29

1000	0.22 (13%)	0.82 (44%)	29

### 3.4. Example parameter space

**Figure 5.**
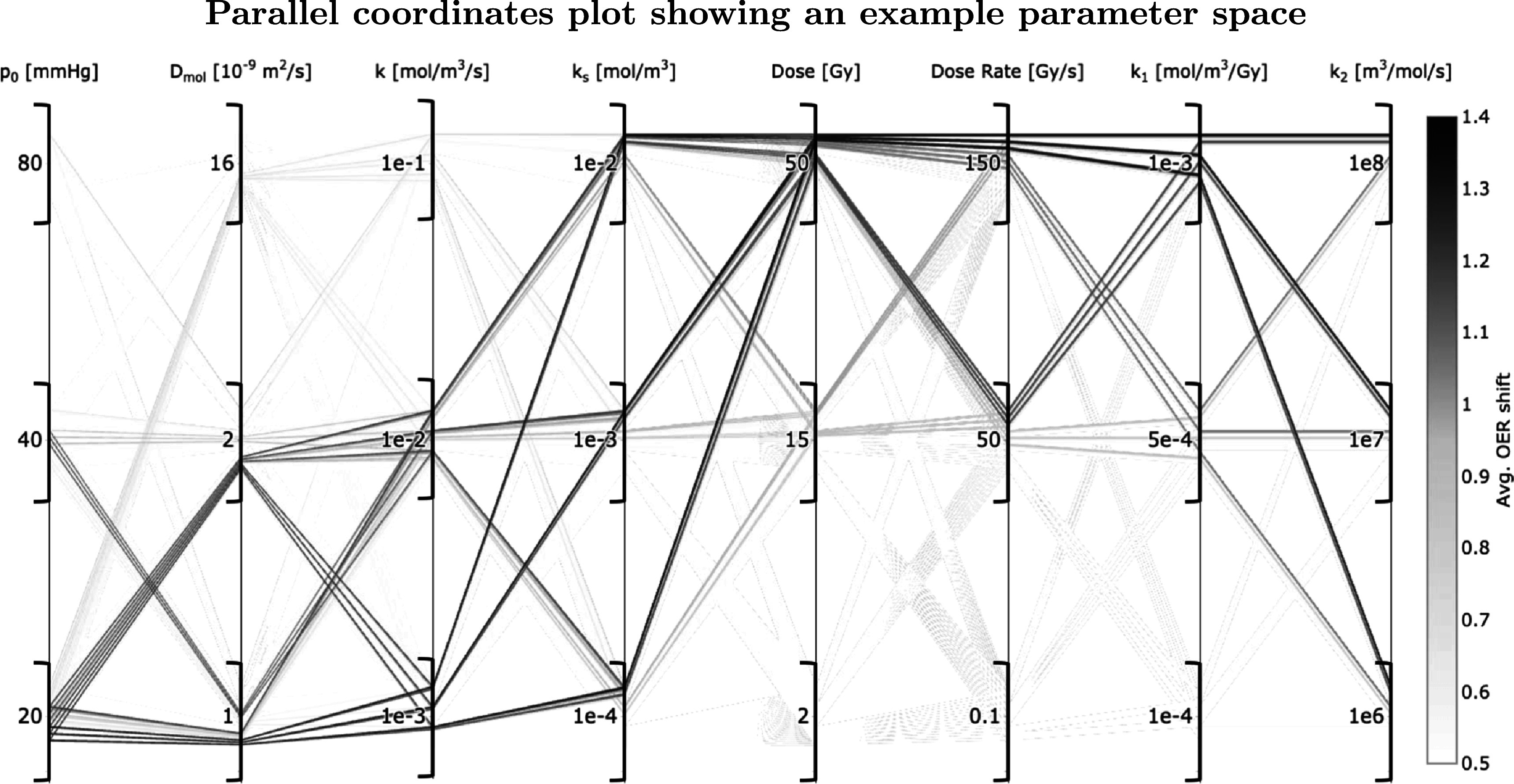
Parallel coordinates plots showing results from the parameter search. Vertical axes represent each parameter, with tested values shown of the scale. Each line represents a simulation run using the combination of parameter values at each axis at which it crosses. The colour scale shows the magnitude of induced OER shift across the nodes.

An example parameter space was constructed using parameter values from literature, summarised in table 2 with references for each parameter. Three values of each parameter were tested in combination giving a total of 6561 simulations. The results are given in figure 5 as a parallel coordinates plot. Data points are mapped across multiple vertical axes with a line (Watts and Crow 2019). Here, an eight-dimensional space represents the eight variable parameters (*p*_0_, *D*_mol_, *k*, *k*_*s*_, *k*_1_, *k*_2_, dose and dose rate,). Each is assigned a *y*-axis, with its respective tested values forming the scale. Each line across the axes represents a single simulation run using a specific combination of tested values. The colour scale of the lines indicates the magnitude of the dependent variable—in this case, the average OER shift across the nodes, with a maximum value (from fully oxygenated to anoxic) of 1.6. Greater shifts indicate a greater decrease in radiosensitivity and are shown by darker lines.

The greatest OER shifts were ∼1.3 (close to the maximum possible shift of 1.6). The maximum ΔOER was measured for the lowest tested values of diffusivity and capillary tension (*D*_mol_ = 1 × 10^−9^ m^2^ s^−1^, *p*_0_ = 20 mmHg), lowest metabolic consumption rate (*k* = 10^−3^ mol m^−3^ s^−1^, *k*_*s*_ = 10^−2^ mol m^−3^), highest radiolytic reaction constants (*k*_1_ = 110^−3^ mol m^−3^ Gy^−1^, *k*_2_ = 10^8^ m^3^ mol^−1^ s^−1^) and highest dose and dose rate (50 Gy, 150 Gy s^−1^). All three tested values of *k*_2_ made up the three highest measured OER shifts, and all values of *k*_*s*_ appeared in the highest nine shifts, with no change in the remaining parameters. This gives an indication of the parameters with the least impact on this measured outcome.

Figure 5 shows that in this parameter space, ΔOER generally increased with increasing dose and dose rate, as expected. No significant shifts were observed for the lowest tested dose rate of 0.1 Gy s^−1^, or the lowest dose of 2 Gy. Even for the maximum dose rate of 150 Gy s^−1^ a dose of ≳15 Gy was required for ΔOER ≳1.0. For the maximum dose of 50 Gy a dose rate of ≳50 Gy s^−1^ was required. This suggests both dose and dose rate are important in achieving a sparing effect.

It is also clear from the stark difference in colour scale along the *k*_1_ axis that this parameter has a strong impact on ΔOER. No significant shift was measured for *k*_1_ = 1 × 10^−4^ mol m^−3^ Gy^−1^—even at maximum dose and dose rate values, ΔOER did not exceed 0.4. Conversely for *k*_2_, the spread of darker lines along this axis shows that large shifts were measured at all tested values, indicating the relative insignificance of this parameter in the range of tested values.

The first four parameters in figure 5 determine the initial oxygen concentration in the system, as well as the speed of recovery after radiation. The greatest shifts here were generally observed for low values of *p*_0_ and *D*_mol_, indicating greater sparing for cells that are more hypoxic at the time of radiation. However, significant shifts also demanded low values of *k* (i.e. a lower metabolic consumption rate). It could therefore be interpreted that no further sparing is available once the cells become too hypoxic, which could be the case for tumour tissue.

To investigate this hypoxic limit further and model a more tumour-like case, the system length, *L*, was increased from 20 to 100 *μ*m to reflect an increased inter-capillary distance (from 40 to 200 *μ*m) and reduced blood flow in a hypoxic tumour core (Secomb *et al* 1993). Simulations were run using the steady state and radiolytic parameters which gave the largest ΔOER (∼1.3) in the parameter space in figure 5. It was found for even the highest dose and dose rate values tested, ΔOER did not exceed 0.2 for *L* = 100 *μ*m, and so no significant shift was observed.

As large shifts were observed for all tested values of *k*_*s*_, it is likely this is relatively insignificant for this parameter space, which would suggest a zero-order approximation for metabolic rate may be a reasonable assumption. However, if we constrain the parallel coordinates plot to the lowest values of *p*_0_ and *D*_mol_ and high values of *k* (≥ 10^−2^ mol m^−3^ s^−1^), significant shifts are only observed for the highest value of *k*_*s*_. This suggests *k*_*s*_ becomes more important at low concentrations.

To compare with Zhou *et al*’s (2020) prediction that a clinical effect is observed for a 3% relative change in radiosensitivity, the maximum measured values of ΔOER correspond to relative shifts of ∼50%. Shifts of ≥3% were measured for approximately 1/6 of the total parameter space. This included only dose rates ≥50 Gy s^−1^, but all values of other parameters.

## 4. Discussion

A proof-of-concept model of oxygen diffusion and reaction in cells has been presented. This provides a tool to aid in the investigation of radiolytic oxygen depletion, which arises from ultra-high-dose-rate irradiation of cells and is hypothesised to result in normal tissue sparing. The model is based on underlying principles of oxygen kinetics, using Fick’s laws of diffusion to describe oxygen transfer from the blood supply into cells, and of Michaelis–Menten-style kinetics to model metabolic oxygen consumption in cells—an approach that is generalised for any cellular oxygen concentration, but not often adopted in similar models due to the complexities involved in obtaining a solution. Here, a numerical solution was obtained using a cellular automaton. Similar reaction-diffusion models have been solved using various methods of finite difference (Pratx and Kapp 2019a). However, due to the nature of error propagation in this approach this type of solution can often lead to instabilities and cannot as easily be partitioned into individual processes (Crank 1975). The model can be expanded into two or three dimensions to account for the geometrical divergence of the oxygen flux diffusing from the capillary, but at the expense of increased computational effort.

A number of assumptions have been made in the development of this model. Firstly, by fixing the boundary condition at *x* = 0, we ignore the possibility that blood is affected by radiation. Although this limits investigation into the perfusion-limited hypoxia scenario, the main focus of this present work is regarding how diffusion-limited hypoxia could be subject to the FLASH effect. Secondly, it was assumed that the Henry’s Law constant used to convert dissolved oxygen concentrations into partial pressures was that of pure water (Blanch and Clark 1997). If the salt solution concentration is increased, the Henry’s Law constant could differ by a factor of 2. However, by overestimating the amount of dissolved oxygen this makes the FLASH effect less likely, and so provides a worst-case scenario. Thirdly, it is assumed that the relationship between OER and oxygen tension applies to both tumour and normal tissue in the same way (Grimes and Partridge 2015), and that this relative change in radiosensitivity can be used as an indicator for potential tissue sparing.

We have also assumed lumped reactions for the radiation-induced consumption of oxygen. Although there is a vast literature on the chemistry of radiolysis (Wardman 2020), this concentrates on individual reactions which contribute to the consumption of molecular oxygen, while rarely considering the total consumption. Since we are interested in the total consumption in this present work, we have not sought to model individual reactions at this stage. Investigations into the dependence of the radiolytic model on oxygen concentration were done, as there has been variation in similar models either using a zero-order (Pratx and Kapp 2019a), first-order (Petersson *et al* 2020), or two-stage approach (Zhou *et al* 2020). Similar results were obtained for the two-stage and zero-order cases using various literature values for the radiolytic rate constants. The dose-dependent production of radiolytic species in the two-stage model drives the depletion of oxygen in the second stage, thereby tending towards the zero-order case where the rate of depletion is independent of initial oxygen concentration (Weiss *et al* 1974). However, a zero-order reaction is unrealistic, and checks in the code were necessary to ensure the concentration did not become negative. Further investigations can determine whether this assumption may hold at very low oxygen levels. Values for the depletion constant in the first-order case are even less well-established, and large variation in the depletion levels was found for the range tested. This therefore may not be the most appropriate model to use until more conclusive radiolytic depletion measurements are made. In any case, the choice of radiolytic model remains a flexible feature within the model allowing for further investigation.

The model has been built using a number of parameters involved in the oxygen depletion process, including those to do with dose delivery, radiochemical depletion kinetics and inherent biological features of the tissue. Here we have assessed the impact of some of these parameters and used ranges of values from literature to demonstrate the extent of possible outcomes. It was found just from the biological parameters that there is large variation in possible steady-state conditions from literature, even before radiation is considered, with nodes ≳10 *μ*m from the capillary being in a state ranging from hypoxia to physoxia depending on the metabolic consumption (figure 2) (McKeown 2014). These parameters are important to establish the initial oxygen conditions which determine the potential for sparing. Precise measurements of the oxygen diffusion and metabolic parameters are therefore essential to accurately model oxygen depletion.

Further variation in outcome is obtained when we consider the dose depletion kinetics. For the two-stage depletion model, the range in values for the radiolytic species production constant, *k*_1_, resulted in depletion levels of between ∼3% and ∼30% for the same dose and dose rate (figure 3). This emphasises the importance of an increased understanding of the radiochemistry underlying the FLASH effect—specifically, the leading chemical reactions which drive the total rate of radiolytic oxygen depletion. From the example parameter space given in figure 5, significant values of ΔOER were only measured for *k*_1_ ≥ 0.0005 mol m^−3^ Gy^−1^, but for all tested values of *k*_2_, further corroborating this idea that oxygen depletion is driven by the production of radiation-induced species in the first stage (Weiss *et al* 1974). Establishing accurate values for the radiolytic depletion rate should be a focus for future experimental work.

Arguably the most important parameter in the context of FLASH radiotherapy, the impact of the dose rate on ΔOER was assessed using the model (figure 4). Significant increase in ΔOER was observed for increasing dose rates up to ∼100 Gy s^−1^, but with no further increase at 1000 Gy s^−1^. The saturation of average ΔOER with dose rate supports the suggestion that a FLASH threshold may exist within 50–150 Gy s^−1^ (Vozenin *et al* 2018, Spitz *et al* 2019, Zhou *et al* 2020), as well as the increase in FLASH effect between 30 and 100 Gy s^−1^ observed by Montay-Gruel *et al* (2017). However, the results from the parameter search in figure 5 show that even at these high dose rates, no significant shift was observed without a sufficiently high dose (∼15 Gy). The results from these investigations suggest that the 3% minimum change in radiosensitivity to observe a clinical effect, used by Zhou *et al* (2020), may be an underestimate. Although dose rates of ≳50 Gy s^−1^ were required to meet this threshold, all values of the remaining parameters, including a dose of 2 Gy, were also sufficient. The largest relative shifts measured here reached up to ∼50% (ΔOER of ∼1.3). Compared to the maximum value of 61.5% (ΔOER of 1.6) at which fully oxygenated cells become anoxic, these large shifts may further demonstrate the feasibility of FLASH-induced oxygen depletion for a biologically-relevant range of parameter values.

From both early and recent literature there exists a threshold in initial oxygen tension for a FLASH effect where we can achieve nearly full depletion of oxygen from a given radiation dose (Vozenin *et al* 2019). Breakpoint survival curves have shown that a sparing effect only occurs when there is sufficiently high dose, or sufficiently low initial oxygen tension (Nias *et al* 1969, Berry and Stedeford 1972). The saturating curve of relative radiosensitivity plotted as a function of oxygen tension shows that a given level of depletion will produce a greater change in radiosensitivity if the initial oxygen level is on the edge of the shoulder of the curve (at ∼20 mmHg) (Hall and Giaccia 2012). For cells that are too well-oxygenated, the radiosensitivity is too saturated to result in any shift, and for cells that are initially too hypoxic, there is less potential for a shift in radiosensitivity before complete anoxia is achieved (it is assumed that the latter is the reason for a lack of FLASH effect in already-hypoxic tumour tissue). It was found here that low values of capillary tension and diffusivity, resulting in low oxygen tensions, produced greater OER shifts. However, for the highest metabolic consumption rates considered, leading to further lowering of the initial oxygen tension, no significant shifts were observed. This may indicate the limiting case for sparing availability, thus supporting this concept.

To further investigate this, and a potential differential effect in tumours compared to normal tissue, the inter-capillary distance was increased from 40 to 200 *μ*m to reflect the condition in a particularly hypoxic tumour core with reduced blood flow and thus oxygenation (Secomb *et al* 1993, Muz *et al* 2015). The parameter combination from figure 5 which gave the greatest OER shift (for an inter-capillary distance of 40 *μ*m) was tested again using a distance of 200 *μ*m, resulting in a 85% decrease in ΔOER from 1.3 to 0.2. Increased inter-capillary density in hypoxic tumours may therefore contribute to their differential response to FLASH irradiation observed in literature (Favaudon *et al* 2014).

It is of interest in future studies to compare specific parameters for tumour and normal tissue to explore a differential FLASH effect. However, there is a lack of well-defined and validated data for each case. For example, reported values of oxygen consumption rates and diffusion constants in tumours and normal tissue span a similar range (Vaupel *et al* 1989, Pogue *et al* 2001, Patzer 2004). Tumour heterogeneity (both for different types of tumour and within a tumour), complex vasculature, and a lack of data mean that the model parameters are not well-defined. The model therefore drives the need for validated tissue-specific data to verify specific cases where a FLASH effect is or is not apparent. Regardless, it is generally established that tumours are usually more hypoxic than normal tissue (Muz *et al* 2015). The results here predict enhanced sparing for tissue at relatively low oxygen levels (such as the skin) but not for severely hypoxic regions (such as in many tumours), particularly those with high metabolic rates or intercapillary distance.

A Michaelis–Menten-style approach was used to model metabolic oxygen consumption, due to the generality in describing metabolic effects from a range of oxygen tensions, with the metabolic saturation point being consistent with the concept of hypoxia. For *k*_*s*_ ≫ *C*, the kinetics become first-order in concentration, and for *k*_*s*_ ≪ *C*, become zero-order. Results here showed that *k* has a significantly greater impact on ΔOER than *k*_*s*_, indicating that a zero-order approach may be an appropriate assumption in most cases (Place *et al* 2017). However, as the results were constrained to low oxygen concentrations, the importance of *k*_*s*_ became apparent. Several recent reaction-diffusion models assume zero-order metabolic consumption (Gerlee and Anderson 2010, Grimes *et al* 2014, Aleksandrova *et al* 2016, Pratx and Kapp 2019a), and so caution should be taken in describing metabolic consumption at low oxygen levels (particularly for FLASH-related investigations where the concept of hypoxia becomes important).

A further point in which this present work deviates from recent models is the use of an effective oxygen diffusivity. The molecular diffusivity often adopted does not consider the porous nature of tissue, and assumes oxygen diffuses freely. Using first-approximation values of tortuosity and voidage, it was found here that the effective diffusivity varies from the molecular diffusivity by up to an order of magnitude, and results in steady-state oxygen levels reduced by more than 50% at 20 *μ*m from the capillary (figure 2). Models which use the molecular diffusivity may therefore assume the oxygen tension in cells to be closer to the capillary tension, perhaps overestimating physiological oxygen levels in normal tissue or determining a lower capillary tension than necessary to result in FLASH sparing. For example, Pratx and Kapp (2019a) proposed that only cells which are sufficiently far from a capillary (≳75 *μ*m) would experience a sparing effect from FLASH radiation, thereby hypothesising FLASH sparing to be driven by stem cells in particularly hypoxic niches (Pratx and Kapp 2019b). While our initial results agree that low initial tensions are necessary for a significant sparing effect, it is likely that incorporating tortuosity and voidage into this model of oxygen depletion would decrease predicted rarity of a sparing effect in typical normal tissue.

One of the biggest limitations in models such as this in determining accurate quantitative results for oxygen depletion is the uncertainty of measured parameter values, both from the range of literature values reported and from the uncertainties of measurements themselves. One of the main aims of this study was to demonstrate some extent of this variation and how it may impact predicted results. The model can be used to identify key experimental data which must be acquired to validate FLASH-related hypotheses. For example, radiolytic measurements of oxygen depletion from cell media or serum at different dose rates could determine the radiolytic depletion model and associated rate constants. Oxygen consumption rate measurements at different oxygen concentrations for the tumour or normal tissue cell line of interest could verify the Michaelis–Menten metabolic parameters. General measurements of tumour or normal tissue oxygenation levels *in situ* would be useful to verify the steady state parameters for the specific tissue of interest. Continued FLASH *in vitro* and *in vivo* studies using varied dose rate and dose are required to determine optimal delivery conditions to achieve a FLASH effect. As pre-clinical data develops, these can be fed back to improve the model further and verify results. Well-established, tissue-specific parameters would be ideal to identify areas in the body more or less likely to exhibit FLASH sparing, and to further explore a differential effect between tumour and normal tissue.

Further work using the model may involve further variation of existing parameters to create a more comprehensive parameter space (requiring increased computational effort) with other parameters, such as the node width, tortuosity, voidage etc, included. Additional parameters can also be introduced to explore any volume effects, different vasculature systems or blood flow scenarios, the effect of different radiation pulse sizes, fractionation regimes, or other delivery modalities with variable spatial and timing characteristics. The model has been designed with scope for these kinds of development in mind, and with flexibility to incorporate additional mechanisms if oxygen depletion is found to not provide a full explanation for the FLASH effect.

## 5. Conclusion

We have presented a modelling tool for FLASH-related investigations into radiolytic oxygen depletion. The results from this study recommend that the FLASH effect should not be considered as having a threshold solely in dose rate. For a significant shift in radiosensitivity, it was found here that the dose and radiolytic depletion rates must also be sufficiently high, and a number of biological parameters must combine to give an initial oxygen concentration that is sufficiently (but not excessively) low. A sparing effect is therefore the result of a multi-parameter situation. While FLASH studies have demonstrated the requirement for a low initial oxygen concentration and high dose, we have presented a quantitative tool to determine more precise values for different scenarios. By establishing a clinically-relevant parameter space for FLASH radiotherapy, the model can be used to answer more complex questions surrounding the mechanism underlying the effect, and provide a pathway to patient benefit via biologically-informed, model-based treatment planning.
